# Case of Fracture of the Anterior Inferior Spinous Process of the Ilium

**Published:** 1852-03

**Authors:** Charles W. Ashby

**Affiliations:** Culpepper County, Virginia


*Case of Fracture of the Anterior Inferior Spinous Process of the Ilium.* By CHARLES W. ASHBY, M. D., of Culpepper County, Virginia. [Communicated by Professor Mütter.]

To the Editors of the Medical Examiner:

Gentlemen: I have just received the enclosed interesting letter from my friend, Dr. Ashby, one of the most eminent surgeons of the State of Virginia. As the case reported has never, to my knowledge, been described, I have thought its publication would prove of value to the profession.

Yours faithfully,

Feb. 18th, 1852. THOS. D. MÜTTER.

Professor Thomas D. Mütter:

*Dear Sir:*—Your kind letter requesting me to send you a report of my case of "Fracture of the Anterior Inferior Spinous process of the Ilium," ought to have been replied to long since.

As you say "there is no such case on record," I will endeavor to detail the symptoms of the accident which occurred in my practice, in such a manner as will enable you to judge of the correctness of my diagnosis.

A strong, athletic negro man, 19 years old, was walking rapidly from a spring, in view of an approaching storm, and, as the night was very dark, he stepped into a gully about a foot and a half deep. He had upon his head at the time, an unusually large tub of water. Although he did not fall, he was so disabled as to require the assistance of men to carry him to the house.

There was great loss of power in the right leg, though not entirely deprived of muscular control, except as to elevation. As I could make the limb perform all the natural movements without much pain, and as I could not perceive, after the most careful examination, the slightest distortion, no lengthening or shortening, I decided, very confidently, that there was neither fracture or dislocation. But the boy, who was more than ordinarily intelligent, insisted most strenuously that he heard and felt something give way, not only at the time the accident occurred, but whilst I had been making the examination.

Upon elevating the leg at right angles with the body and letting it down rather suddenly, I now, for the first time, heard a crepitus, I confess, much to my surprise. By this particular movement and *by no other*, the crepitus was so distinct as to be heard, not only by myself again and again, but by all the bystanders. What fracture have I here? was a most natural enquiry. From the history of the case, I was, at first, inclined to suspect, though in a young subject, a fracture of the neck of the bone, and being aware of the various natures, as well as great obscurity of this accident, my mind was directed most anxiously to its investigation. Reasoning by exclusion, I became satisfied that this could not be the fact; there was not a single symptom which is usually present in this accident—there was little or no pain or tumefaction about the joint, and, indeed, not a sufficient amount of irritation to warrant the belief that there was a fracture of any of the large bones, either of the pelvis or leg.

I requested the boy to direct my hand to the spot where he felt the greatest amount of pain. He placed it in his groin, where I detected, for the first time, a good deal of tenderness and tumefaction. Pressing two fingers of my left hand firmly upon this spot, and with my other hand elevating the leg and letting it down as before, I not only heard the crepitus, but I felt distinctly a spiculum of bone moving under my fingers. This manifestation, not *very* painful to my patient, was performed not once, but I may safely say more than twenty times, and invariably with the same result, before I decided *positively* as to the *precise* character of the fracture.

I was surprised to find that I had been poring over this case at the dead hour of night, for more than three hours. I had never heard or read of such an accident, and therefore only those of my professional brethren who know me best, can appreciate the deep anxiety I felt as to the making out a correct, satisfactory diagnosis.

The maxim that "there is nothing new under the sun," often repeated by a distinguished Professor of my Alma Mater, did not fail to make an impression on my mind, and, properly understood, riper years have served only to deepen that impression. I have not the slightest shadow of a doubt, as to the correctness of my diagnosis. As to its novelty, I have the high authority of your name, as well as that of other distinguished surgeons, and yet I am of the opinion that the accident has occurred before, but either has not been recognized, or has not been thought worthy of being recorded.

Whether the process was pulled off by the powerful contraction of the recti muscles, or by the tremendous jar of the head of the bone, thrown, in the act of stepping forward, upon the outer edge of the acetabulum, or by both causes conjointly, I leave for others to decide.

The treatment of this case was as effectual as it was simple, and the result, I think, confirmed the diagnosis.

After flexing the limb, a roller six or eight yards long, was passed firmly around the thigh, so as to control muscular action, then passed firmly over a wet compress, placed over the process, thence around the body and back over the compress and around the thigh again. The boy was placed on his side, and experienced immediate relief, so that he slept soundly the balance of the night. Strict rest was enjoined, and he suffered little or no pain after the bandage was applied. In four weeks he was walking about, but wore the bandage and compress for several months, as, he says, it gave him great support.

I am, with high consideration, yours, &c.

CHAS. WM. ASHBY.

P. S. This accident occurred in Nov., 1842.

